# Glutamate metabolism reprogramming is involved in the inhibitory effect of ellagic acid on rotavirus replication

**DOI:** 10.3389/fcimb.2026.1958706

**Published:** 2026-09-24

**Authors:** Jiangang Zheng, Zhigang Cao, Wafa Yousaf, Abdul Haseeb, Zixuan Zhang, Luxi Wei, Tian Lan, Kang Kang, Haiyun Wu, Hejie Wang

**Affiliations:** 1 School of Public Health, Changzhi Medical College, Changzhi, Shanxi, China; 2 Shanxi Higher Education Institutions of Science and Technology Innovation Plan Platform, Laboratory of Environmental Factors and Population Health, Changzhi, China; 3 Key Laboratory of Environmental Pathogenic Mechanisms and Prevention of Chronic Diseases, Changzhi Medical College, Changzhi, China; 4 Shanxi Provincial Innovation Center for Upper Gastrointestinal Cancer Research and Clinical Translation, Changzhi Medical College, Changzhi, Shanxi, China; 5 Four “Batches” Innovation Project of Invigorating Medical through Science and Technology of Shanxi Province-Key Laboratory of Esophageal Cancer Basic Research and Clinical Transformation, Heping Hospital Affiliated to Changzhi Medical College, Changzhi, China; 6 College of Veterinary Medicine, Shanxi Agricultural University, Taigu, Shanxi, China

**Keywords:** ellagic acid, glutaminease 1, L-glutamate, rotavirus, untargeted metabolomics

## Abstract

**Introduction:**

Rotavirus (RV) is an important zoonotic pathogen that causes acute diarrhea in infants and young animals. There are several limitations in the prevention and control strategies currently, which result in an urgent need to develop innovative natural active substances against RV. This study aims to explore the RV infection preventive mechanism of ellagic acid (EA) and determine whether it exerts its effect by regulating the glutamine-glutamate metabolic axis.

**Methods:**

RV-infected Balb/c mice and IEC-6 cell models were established to conduct mechanistic research by employing untargeted metabolomics, protein immunoblotting (WB), real-time fluorescence quantitative PCR, etc.

**Results:**

The non-targeted metabolomics results revealed that RV infection could disrupt the host's L-glutamate metabolism, and the serum L-glutamate level further decreased after ellagic acid (EA) intervention (*P* < 0.05). The WB results showed that EA could significantly inhibit the protein expression of glutaminease 1 (GLS1) in the jejunum tissue of mice (*P* < 0.001). The pathway rescue test using DON, a broad‑spectrum glutamine antagonist, showed that DON could reverse the inhibitory effect of ellagic acid on RV replication, suggesting that glutamine‑glutamate metabolism contributes to the antiviral action of ellagic acid (*P* < 0.05).

**Discussion:**

RV promotes glutamine catabolism by up-regulating GLS1 expression, thereby increasing the production of L-glutamate, which serves as a critical metabolic substrate supporting virus replication. Ellagic acid downregulates GLS1 expression and effectively disrupts metabolic processes, depriving the virus of essential nutrient inputs required for efficient propagation. Simultaneously, exerting anti-inflammatory and antioxidant activities to alleviate RV-induced intestinal injury. This study has elucidated new metabolic regulatory mechanistic insights of ellagic acid against RV, thereby providing experimental foundation and theoretical support for the application of natural bioactive compounds in RV prevention and control, as well as in the development of targeted antiviral therapeutics.

## 1 Introduction

The rotavirus genus comprises a double-stranded RNA (dsRNA) genome belonging to the Sedoreoviridae family within the Reovirales order (Matthijnssens et al., 2022). Rotavirus is the leading pathogen causing acute diarrhea-related deaths in children under 5 years old worldwide, resulting in approximately 2 million hospitalizations and 700,000 deaths each year, with the vast majority occurring in developing countries (Parashar et al., 2003; Cunliffe and Nakagomi, 2005; Cates et al., 2022; Zhou et al., 2025). Moreover, rotavirus is also a major diarrhea-causing agent in young animals as well, with a prevalence of 7% to 94% in calves worldwide (Hou et al., 2025). The infection rate of swine type A rotavirus (RVA) ranges from 3.3%-67.3%, and despite the availability of commercial vaccines, the prevalence of RVA infection on pig farms remains persistently high (Vlasova et al., 2017).

Currently, Rotarix and RotaTeq vaccines have been included in the immunization programs of 100 countries, but the RV-related mortality rate in underdeveloped countries is still on the rise (Patel et al., 2012; Bonura et al., 2022). In the context of animal disease prevention, commercial rotavirus vaccines for cattle, pigs, and horses are available; however, their application is mainly focused on the immunization of pregnant animals to provide early protection for newborn animals through inducing maternal passive immunity, which results in limited achievement of comprehensive interruption of virus transmission (Carossino et al., 2024; Kumar et al., 2022). Among different strains of rotavirus, type A rotavirus (RVA) is an important pathogen causing acute diarrhea in infants and young animals. It exhibits cross-species transmission potential and carries a zoonotic spread risk. In environments with frequent human-animal direct interaction, it is prone to the emergence of new recombinant strains through genetic recombination, posing a potential threat to public health (Alaoui Amine et al., 2020). Therefore, the development of new anti-rotavirus therapeutic intervention strategies is urgently needed in practice.

Pomegranate peel, a component of traditional Chinese medicine, is often used to treat digestive tract diseases, as evidenced in ancient medical books such as “Puji Fang” and “Leigong Baoshi Lun”. It contains a diverse array of bioactive constituents such as tannins, flavonoids, polysaccharides, and alkaloids. Among these components, tannins represent the predominant class, with anacardic acid and ellagic acid being particularly abundant (Xiang et al., 2022). Anacardic acid is a condensation product derived from ellagic acid and cannot be directly absorbed into the bloodstream through intestinal epithelium; instead, it first undergoes hydrolytic cleavage prior to absorption and utilization in the body (Caballero et al., 2022). The pharmacological effects of ellagic acid mainly include anti-inflammatory, antibacterial, antiviral, and anti-tumor effects, and a natural inhibitor of NF-κB (Aggarwal and Shishodia, 2004; Anitha et al., 2013; Edderkaoui et al., 2008; BenSaad et al., 2017; Jaman and Sayeed, 2018; Acquadro et al., 2020; Cota and Patil, 2023; Yang et al., 2023). Studies have shown that ellagic acid can alleviate diarrhea, so it may have a very high potential value as an anti-RV therapeutic agent (Chen et al., 2020). In our previous study, we established an RV-infected Balb/c mouse model to verify the potential antiviral role of ellagic acid through alleviating intestinal damage (Zheng et al., 2025).

The replication of rotavirus firmly depends on the metabolic processes of host cells, including glutamine/glutamate metabolism, fatty acid and lipid droplet synthesis, and cholesterol homeostasis (Crawford et al., 2012; Mitra et al., 2024; Desselberger, 2024). Ellagic acid (EA) possesses the capability to exert substantial metabolic regulatory effects by regulating glucose homeostasis, lipid metabolism, and intestinal flora (Wang et al., 2024; Leng et al., 2025). Based on this rationale, the present study aimed to employ non-targeted metabolomics and other complementary analytical approaches for comprehensive screening of the host differential metabolites following rotavirus infection and ellagic acid intervention. By enriching and analyzing related metabolic pathways, we sought to determine whether ellagic acid can reverse the host metabolic reprogramming mediated by rotavirus, thereby disrupting the essential components and energy supply required for virus replication. Based on above background, our central hypothesis is that rotavirus infection rewires host glutamine-glutamate metabolic reprogramming by elevating GLS1 expression to supply metabolic substrates for viral replication; ellagic acid may counteract RV propagation partly by suppressing GLS1-driven glutamine catabolism and perturbing this virus-hijacked metabolic axis.

## 2 Materials and methods

### 2.1 Cell lines, compounds, virus, and antibodies

IEC-6 (iCell-r016) cell line was purchased from iCell (China) and cultured in Dulbecco’s modified Eagle’s medium (DMEM, HyClone, USA) containing 10% fetal bovine serum (FBS, BI, Israel) (10% DMEM) and maintained in DMEM containing 2% FBS (2% DMEM) during drug trial treatment.

Ellagic acid (Cat: HY-B0183) and 6-Diazo-5-oxo-L-nor-Leucine (DON, Cat: HY-108357) were purchased from MCE (USA), with 99.75% and 99.98% purity, respectively.

Rhesus rotavirus (MMU 18006, 10^6.25^ TCID_50_/0.1mL) was purchased from ATCC (Cat: VR-1739).

Anti-Actin Rabbit pAb (Cat: GB15003) and goat anti-rabbit secondary antibodies (Cat: GB23303) were purchased from Servicebio (China). Anti-GLS1 Rabbit pAb (Cat: bs-10341R) was purchased from BIOSS (China).

### 2.2 Animal modeling and treatment

Four-day-old Balb/C suckling mice (weight 2.5 g) were purchased from SPF Biotechnology Co., Ltd (Beijing, China). The animals were raised in a controlled environment with a 12-hour light/dark cycle, with free access to food and water. All animal experiments were approved by the Ethics Committee of Changzhi Medical College (DW2025072, March 21, 2025) and were conducted as per international guidelines and regulations. After 3 days acclimatization period, the mice were randomly allocated into 3 groups: Control group, Virus group (10^6^ TCID_50_/20 g), and the Ellagic acid group (100 mg/kg), with 8 mice in each group, half male and half female. Twelve hours after RV infection (10^6^ TCID_50_/20 g), mice were treated with ellagic acid for 3 days. The 0.9% saline solution was used to dissolve the doses of ellagic acid. The control group was given an oral gavage of 0.9% saline equal to that of the other groups. Following euthanasia by CO_2_ inhalation, the intestinal feces were collected, and the jejunum tissues were fixed and cryopreserved following dissection. The CO_2_ euthanasia was performed under controlled parametters are as follows: the chamber volume was 50×37×28 cm, the flow rate was 30% chamber volume/min, the CO_2_ concentration gradually increased to 90% of the chamber volume, and the exposure time was 5 minutes.

### 2.3 Untargeted metabolomics

#### 2.3.1 Sample collection

24 h after the final dose administration, blood was collected into heparin anticoagulant tubes. Gently inverted and mixed the tubes, then let them stand at room temperature for 30 minutes. Centrifuged at 2000 g for 10 minutes to separate the upper plasma sample. Then collected the plasma into a 1.5 mL centrifuge tube, quickly froze it in liquid nitrogen, and stored it at -80 °C for later use. Slowly thawed the serum samples at 4 °C, took 100 μL from each sample and mixed with 400 μL of pre-cooled methanol-acetonitrile solution (1:1, v/v), vortexed for 60 seconds, placed at -20 °C for 1 hour to precipitate proteins, centrifuged at 14000 rcf at 4 °C for 20 minutes, and collected the supernatant for injection.

#### 2.3.2 Chromatography-mass spectrometry analysis

Chromatographic conditions: The samples were separated using an Agilent 1290 Infinity LC ultra-high performance liquid chromatography system (HILIC) column; the column temperature was set at 25 °C with a flow rate of 0.3 mL/min. The composition of the mobile phase was A: water + 25 mM acetic acid ammonium + 25 mM ammonia water, B: acetonitrile; the gradient elution program was as follows: 0-0.5 min, 95% B; 0.5–7 min, B changed linearly from 95% to 65%; 7–8 min, B changed linearly from 65% to 40%; 8–9 min, B maintained at 40%; 9-9.1 min, B changed linearly from 40% to 95%; 9.1–12 min, B maintained at 95%; during the entire analysis, the samples were placed in the 4 °C automatic sampler. To avoid the influence caused by fluctuations in the instrument detection signal, the samples were analyzed continuously in a random order. QC samples were inserted into the sample queue to monitor and evaluate the stability of the system and the reliability of the experimental data.

Mass spectrometry conditions: The samples were collected using an AB Triple TOF 6600 mass spectrometer for the first and second spectra. The detection modes were electrospray ionization (ESI) positive ion and negative ion. The ESI source parameters were as follows: auxiliary heating gas 1 (Gas1): 60, auxiliary heating gas 2 (Gas1): 60, ionization curtain gas (CUR): 30 psi, ion source temperature: 600 °C, spray pressure (ISVF): ± 5500 V (positive and negative modes); the detection range of the first mass-to-charge ratio: 60–1000 Da, the detection range of the sub-ion of the second spectrum: 25–1000 Da, the cumulative time of the first mass spectrum scanning: 0.2 s/spectrum, the cumulative time of the second mass spectrum scanning: 0.05 s/spectrum. The second mass spectrum was obtained using the data-dependent acquisition mode (IDA), and the peak intensity value screening mode was used. The defocusing voltage (DP): ± 60 V (positive and negative modes), collision energy: 35 ± 15 eV. The IDA settings were as follows: dynamic exclusion range of isobaric ions: 4 Da; each scan collected 10 fragment spectra.

### 2.4 Western blot analysis

The different protein expression levels in the jejunum tissue (50 mg) were detected by WB. The tissues were lysed with RIPA buffer containing 1 mM protease inhibitor and 1 mM phosphatase inhibitor and collected using a cell scraper. Total tissue protein was extracted, and protein concentration was determined using the BCA protein assay kit (Beyotime Biotechnology, Jiangsu, China). An equal amount of cell lysate was separated on a 10% SAL-polyacrylamide gel and transferred to a tailored polyvinylidene fluoride (PVDF) membrane according to the molecular weight of the proteins. Then the membrane was blocked with 5% non-fat dry milk mixed in Tris-buffered Tween 20 (TBST) for 2 h at 25 °C. Subsequently, the membrane was incubated with the following primary antibodies overnight at 4 °C: Anti-Actin Rabbit pAb (1:3000) and Anti-GLS1 Rabbit pAb (1:1000). Then, the membrane was washed with TBST three times, and incubated the PVDF membrane with goat anti-rabbit secondary antibodies (1:3000) at 25 °C for 2 h. Finally, the target protein was detected by an enhanced chemiluminescence system (Boster, China). Densitometric values of protein bands were quantified by ImageJ software. The intensity of each target-protein band was normalized to the intensity of the corresponding β-Actin loading-control band within the same lane for relative quantitative analysis.

### 2.5 RNA extraction and quantitative real-time PCR

The IEC-6 cells were cultured in 6-well plates, infected with 100 TCID_50_/mL RV (2 mL/well) for 1.5 h. The supernatant was discarded, and fresh medium with 50 μM/L ellagic acid, 10 μM/L DON, and ellagic acid (50 μM/L)+DON (10 μM/L) was added for further incubation in a 5% CO_2_ 37 °C cell incubator for 24 h. Meanwhile, the Control group and Virus group were applied and incubated for 24 h and 48 h. Total RNA was extracted from the cell samples according to the Trizol protocol (Invitrogen, Carlsbad, CA, USA). The RNA concentration and purity were evaluated by Eppendorf BioPhotometer D30 (Eppendorf, USA). The complementary DNA was synthesized using PrimeScript^®^ RT Master Mix kit with gDNA Eraser (TaKaRa, Dalian, China) according to the manufacturer’s protocol. RT-qPCR was performed by using a 7500 Real Time PCR System (ABI, USA). Absolute RT-qPCR was applied to determine the RV VP6 gene copies; a standard curve was generated using serially diluted PCR amplification product. The primer sequence for the RV VP6 gene is as follows: F 5’-AGCGGTAGCGGCGTTATTT-3’, R 5’-ACTGGTCCAACTGGTATCGC-3’.

### 2.6 Statistical analysis

All data were presented as Mean ± SEM. Data were analyzed using GraphPad Prism™ software 5.0 (GraphPad Software, Inc., California, USA). One-way analysis of variance (ANOVA) followed by a Dunnett’s post-test was used to determine the difference between the groups. All groups are compared with the Virus group, * *P* < 0.05, ** *P* < 0.01, *** *P* < 0.001. For **Figure 1**, inter-group differences are denoted by letters, groups sharing identical letters are not statistically different. GraphPad Prism5 was also used to determine the difference using One-way ANOVA followed by a Tukey’s post-test with *P* < 0.05 as the significance cut-off value.

**Figure 1:**
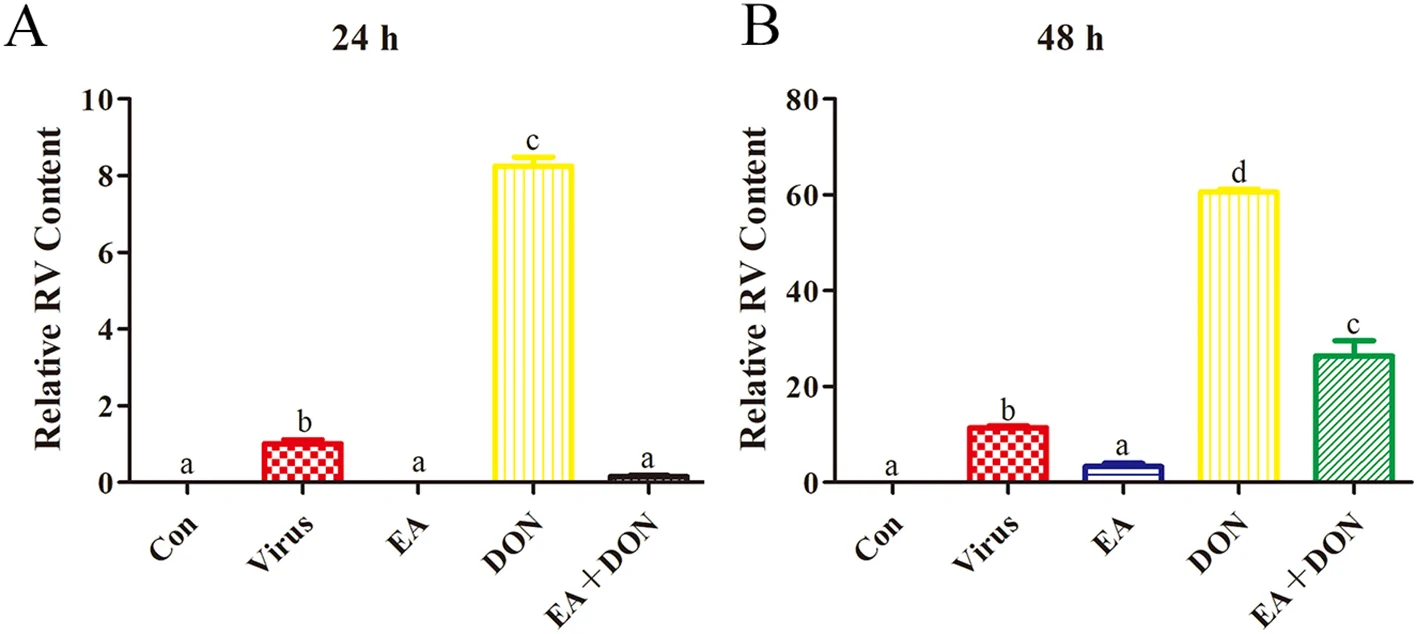
Anti-RV activities of Ellagic acid and DON detected by qPCR. **(A)** IEC-6 Cells were incubated with 100 TCID_50_/mL RV for 1.5 h, then EA (50 μM/L), DON (10 μM/L), and EA+DON were added respectively, and cultured for 24 h. **(B)** IEC-6 Cells were incubated with 100 TCID_50_/mL RV for 1.5 h, then Ellagic acid (50 μM/L), DON (10 μM/L) and EA+DON were added respectively and cultured for 48 h. Lowercase letters a, b, c, d indicate significant differences ((P<0.05), Tukey’s post‑hoc test). Groups with identical letters are not significantly different.

## 3 Results

### 3.1 Screening of differential metabolites in serum

The volcano plot analysis revealed that compared with the control group, the serum of mice in the virus group showed significant upregulation (FC > 2, *P* < 0.05) and significant downregulation (FC < 0.5, *P* < 0.05) of metabolites (**Figure 2A**). Similarly, pairwise comparison between virus and EA-treated mice also identified numerous metabolites with significantly changed abundance (FC > 2 or < 0.5, *P* < 0.05) (**Figure 2B**). Principal component analysis (PCA) was used for inter-group difference analysis. The PCA analysis results showed that the samples of the control group, virus group, and EA treatment group all presented clear clustering separation (**Figures 3C, D**), indicating that both RV infection and EA treatment can significantly affect the metabolic profile characteristics of mouse serum.

**Figure 2:**
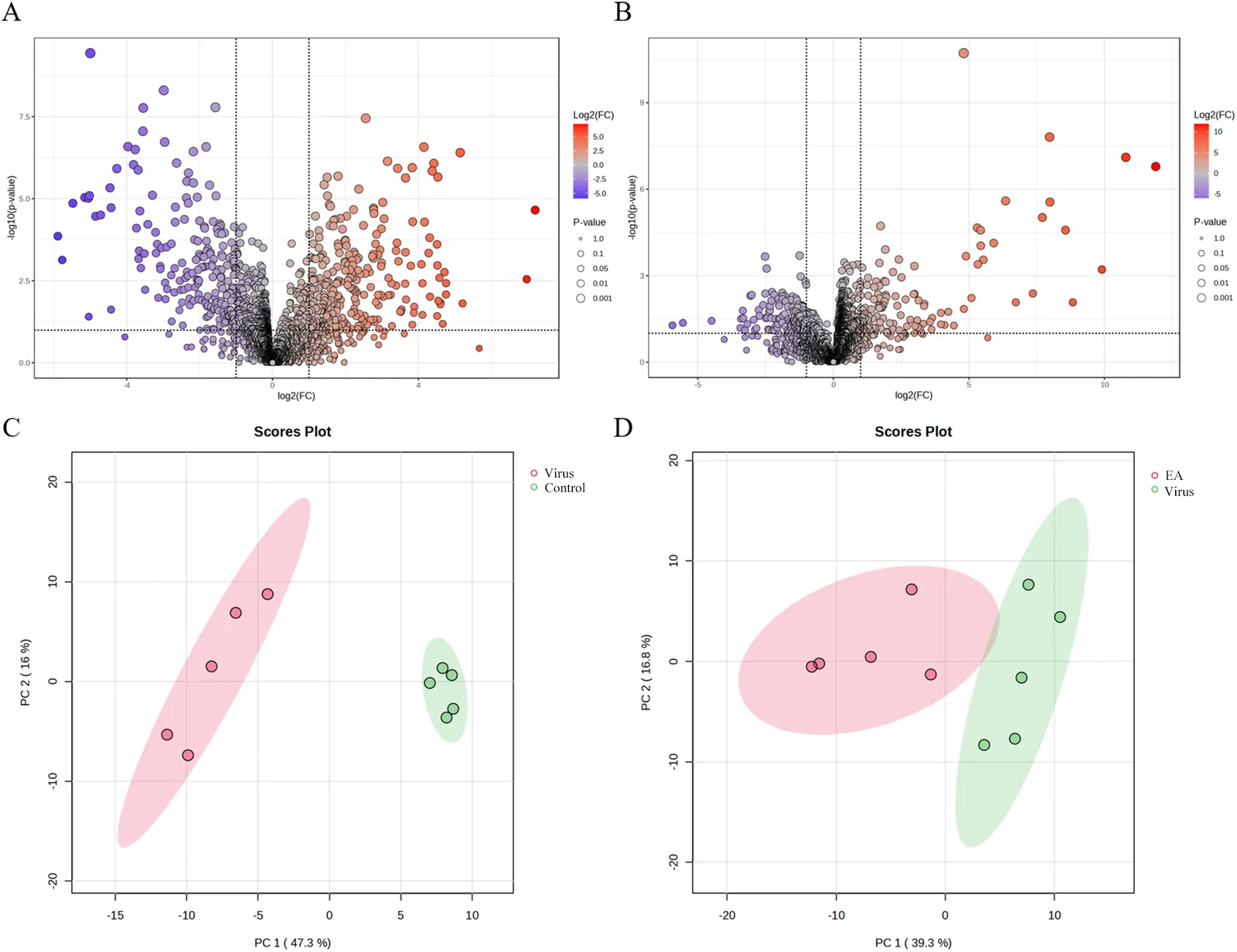
Volcano plot of inter-group differential metabolites in plasma. **(A)** Volcano plot of differential metabolites between the control group and the virus group; **(B)** Volcano plot of differential metabolites between the virus group and the EA treatment group; **(C)** PCA analysis between the control group and the virus group; **(D)** PCA analysis between the virus group and the EA treatment group.

**Figure 3:**
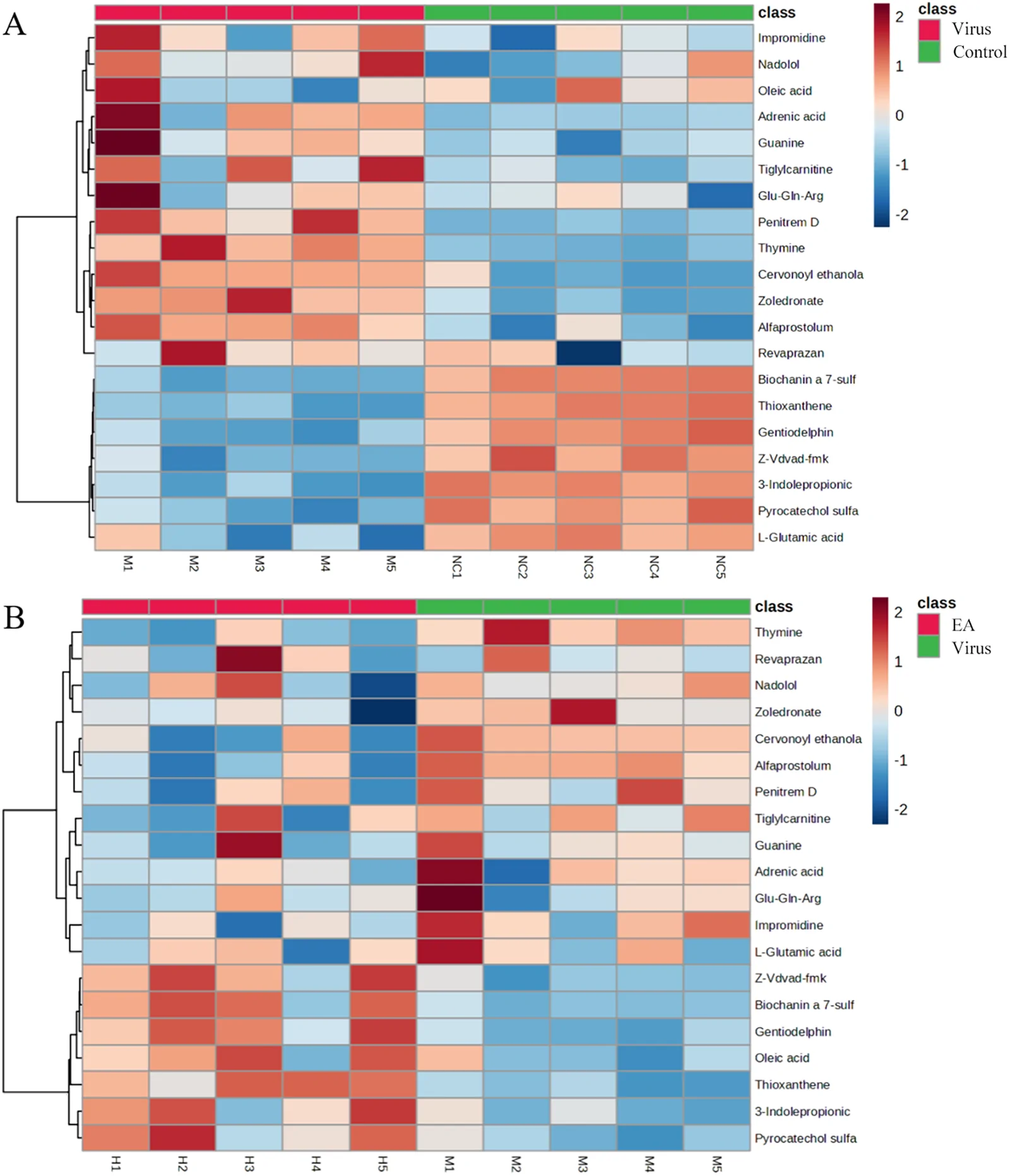
Clustering analysis results of differential metabolites. **(A)** Clustering analysis between the control group and the virus group; **(B)** Clustering analysis between the virus group and the EA treatment group.

### 3.2 Cluster analysis of differential metabolites

The clustering analysis of inter-group differential metabolites showed that compared with the control group, the metabolite contents in the serum of mice in the virus group were significantly increased (*P* < 0.05) for Guanine, Thymine, Tiglylcarnitine, Adrenic acid, Zoledronate, Glu-Gln-Arg, Revaprazan, Alfaprostolum, Cervonoyl ethanolamide, Penitrem D, Impromidine, Nadolol, etc. (**Figure 3A**), while the contents of Biochanin A 7-sulfate, Thioxanthene, Gentiodelphin, Z-Vdvad-fmk, 3-Indolepropionic, Pyrocatechol sulfate, L-Glutamic acid, etc. were significantly decreased (*P* < 0.05) (**Figure 3A**). After EA treatment, the changes in the above differential metabolites showed a significantly reversed trend compared with the virus group (**Figure 3B**). This suggests that EA intervention may exert protective effects by regulating the expression of inflammation-related metabolites such as Guanine, Thymine, Tiglylcarnitine, Adrenic acid, etc., and other differential metabolites induced by RV infection, thereby improving the inflammatory response and metabolic disorders of the body.

### 3.3 Analysis of differential metabolites via KEGG pathways

The KEGG pathway analysis results showed that the differential metabolites between the control group and the virus group were significantly enriched in pathways such as ABC transporters, Bile secretion, Tryptophan metabolism, Pyrimidine metabolism, and Protein digestion and absorption (*P* < 0.05); while the differential metabolites between the virus group and the EA treatment group were significantly enriched in pathways such as ABC transporters, Protein digestion and absorption, and Central carbon metabolism in cancer (*P* < 0.05) (**Figure 4**). This indicates that RV and EA may regulate metabolic pathways such as ABC transporters, thereby altering the content of corresponding metabolites and influencing the disease progression.

**Figure 4:**
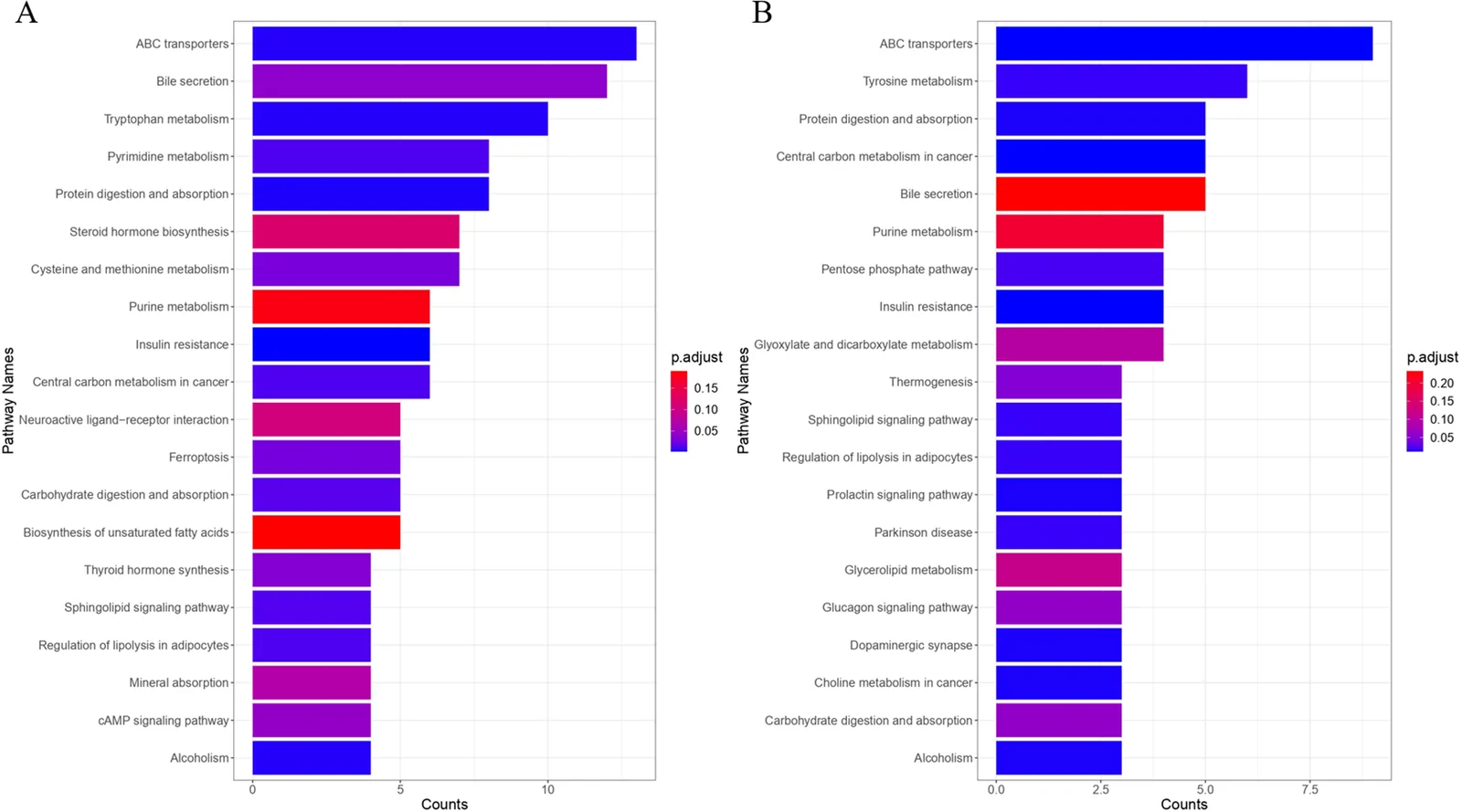
Results of KEGG pathway analysis for differential metabolites. **(A)** KEGG pathway analysis between the control group and the virus group; **(B)** Clustered KEGG pathway analysis between the virus group and the EA treatment group.

### 3.4 DON reversed the anti-RV activity of ellagic acid

The expression of GLS1 protein in the jejunum was detected by WB. Compared with the control group, RV induced the significant (*P* < 0.001) production of GLS1 protein. Compared with the virus group, EA significantly decreased (*P* < 0.001) the expression of GLS1 protein (**Figure 5**). Following 24 h and 48 h treatment of RV-infected IEC-6 cells with ellagic acid and DON, the viral load was analyzed by qPCR to determine the anti-RV efficacy. Compared with the control group, at 24 hours after infection, the virus group only showed focal mild cytopathic effects, with a small number of contracted clusters forming small lesions. At 48 hours after infection, the number of lesion foci increased, the area expanded, a large number of cells contracted and became round, and aggregated, and the integrity of the cell monolayer was partially disrupted. Compared with the virus group, ellagic acid could alleviate the cytopathic effects caused by the virus, while the combination of EA and DON reversed this phenomenon (**Figure 6**). Compared with the virus group, ellagic acid significantly (*P* < 0.05) reduced viral load at 24 h and 48 h. Compared with the EA group, after treatment with ellagic acid combined with DON, the anti-RV activity of ellagic acid was reversed (**Figure 1**).

**Figure 5:**
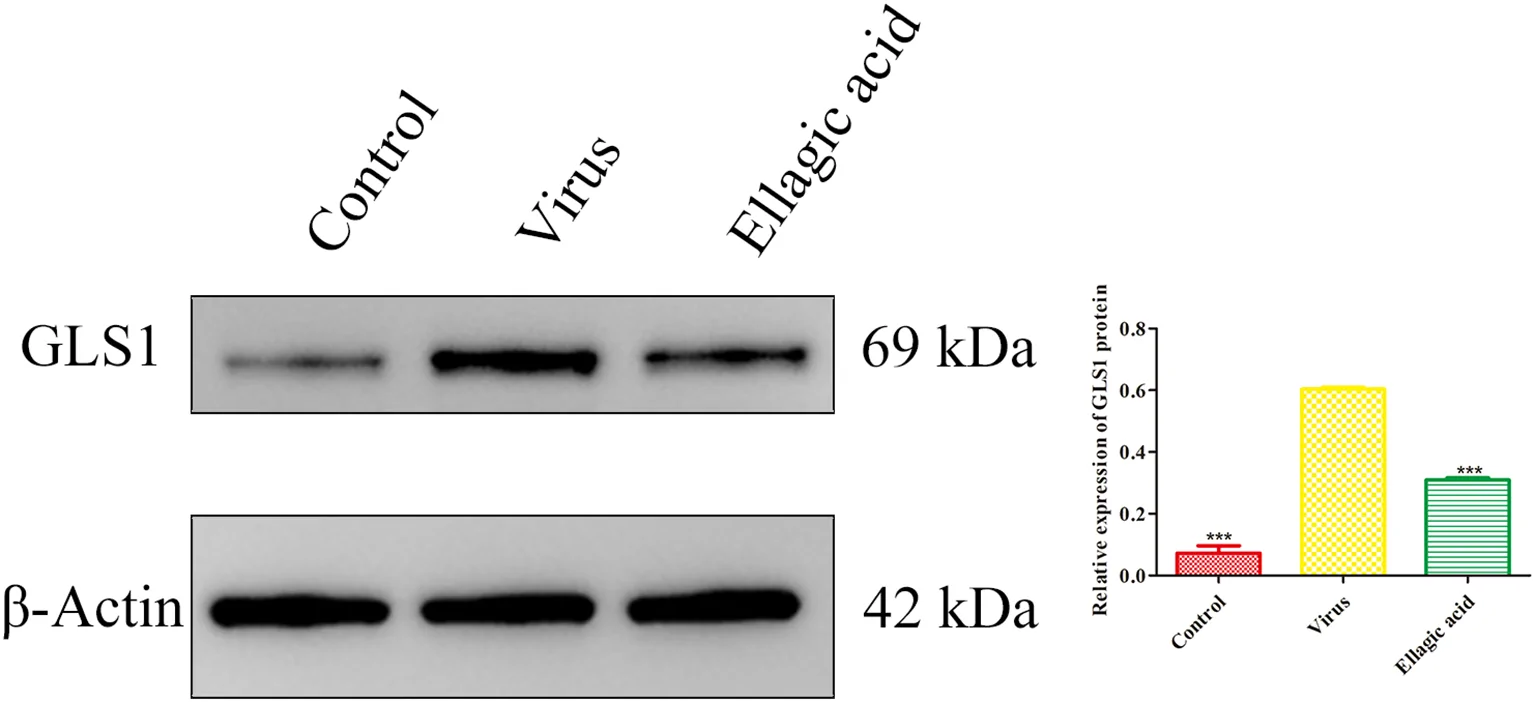
The expression of GLS1 proteins in the jejunum was detected by Western blot. Original blots are presented in Supplementary Figure 1.

**Figure 6:**
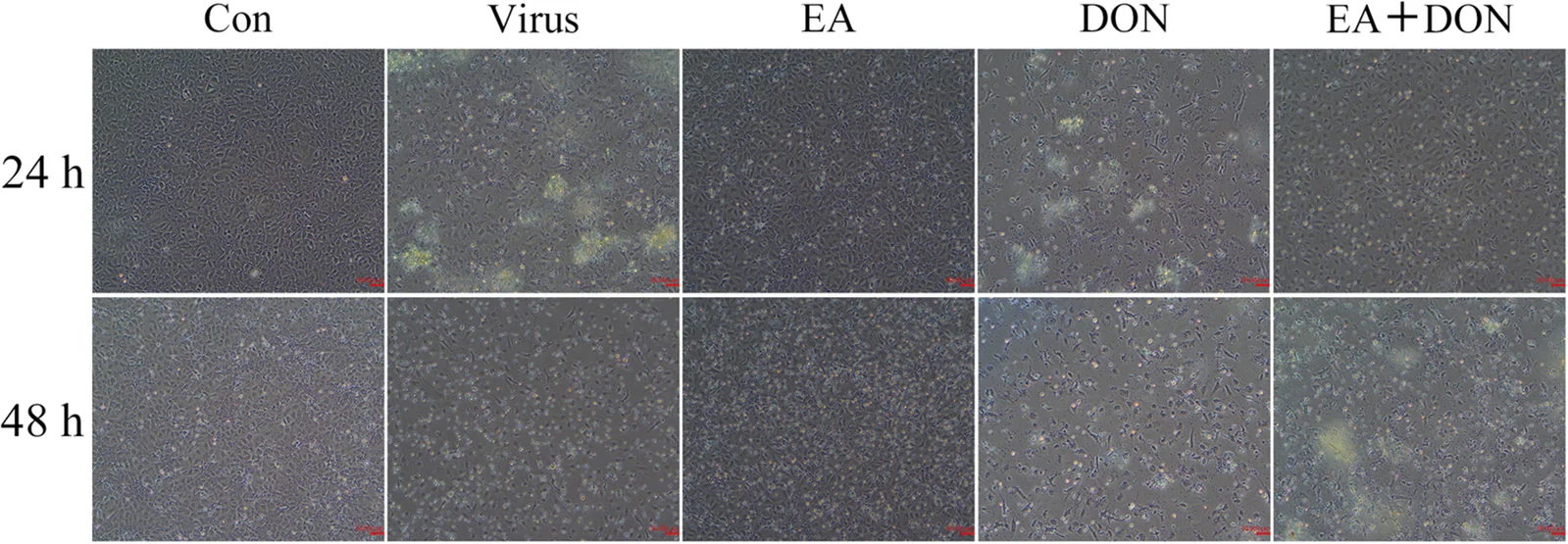
The morphology of IEC-6 cells after 24 h and 48 h of treatment. **(A)** Control group, **(B)** Virus group, **(C)** EA group, **(D)** DON group, **(E)** EA+DON group.

## 4 Discussion

Rotavirus (RV) is the core pathogen causing acute diarrhea in infants, young animals, and young livestock, presenting hazards of high incidence and zoonotic nature, posing a serious threat to public health security and the livestock breeding industry (Parashar et al., 2003; Cunliffe and Nakagomi, 2005; Vlasova et al., 2017; Cates et al., 2022; Hou et al., 2025; Zhou et al., 2025). Currently, the preventive and control strategic therapeutic application like vaccines and intervention measures against RV in clinical and breeding fields, still have limitations, such as protective efficacy, application scenarios, and inability to completely block viral infection and intestinal damage. Developing safe and efficient new natural active substances against RV has significant research value and application significance (Patel et al., 2012; Bonura et al., 2022; Kumar et al., 2022; Carossino et al., 2024). The previous network pharmacology study of this research has confirmed that the natural active component, ellagic acid, can specifically bind to the TLR4 protein, effectively alleviating intestinal tissue damage induced by RV infection, and has clarified that ellagic acid has potential anti-RV infection activity. However, the deep molecular metabolic mechanism regulating RV infection by ellagic acid has not yet been elucidated (Zheng et al., 2024, 2025). Based on this, this research relies on non-targeted metabolomics technology to systematically explore the interaction between ellagic acid and RV infection, focusing on host metabolism, and analyzing the novel mechanism of ellagic acid’s anti-RV effect, providing a new theoretical basis for targeted nutritional intervention and drug development against RV infection.

The non-targeted metabolomics analysis showed that RV infection can lead to a significant decrease in the content of L-glutamic acid in the blood serum of the body, suggesting that the RV-induced host L-glutamic acid-glutamine metabolic disorder. L-glutamic acid is a key functional amino acid in the body, which can be synthesized through the α-ketoglutarate transamination reaction or obtained through dietary protein hydrolysis (Brosnan and Brosnan, 2013). It is the basic raw material for protein synthesis, the primary energy substrate for intestinal epithelial cells, and an essential nutrient for intestinal health (Tomé, 2018). L-glutamic acid is also involved in the synthesis of neurotransmitters such as dopamine and γ-aminobutyric acid, maintaining normal metabolism and immune function of the body (Xue and Field, 2011; Cruzat et al., 2018; Andersen, 2025). Under normal physiological conditions, L-glutamic acid is mainly stored in the body in the form of a protein-bound state, and a small amount is distributed in tissues and body fluids in the free anion state. It can be reversibly converted into glutamine, α-ketoglutarate, and glutathione through glutamine synthetase and glutaminease, achieving a dynamic balance of energy reserve and substance metabolism in the body and enabling rapid mobilization and transportation according to the body’s metabolic needs (Andersen, 2025). Viral reprogramming of amino acid metabolism is a key strategy for hijacking host cells and completing its own replication and proliferation, and the remodeling of the glutamine-L-glutamic acid metabolic axis plays a core regulatory role in the process of intestinal virus infection (Mitra et al., 2024).

RV infection can significantly upregulate the activity of Glutaminase 1 (GLS1) in intestinal epithelial cells, accelerating the extensive catabolism of glutamine and promoting the production of large amounts of L-glutamic acid (Mitra et al., 2024). The accumulated L-glutamic acid can further participate in the tricarboxylic acid cycle, providing ATP energy and various biosynthetic precursors for cellular metabolism, and providing sufficient energy support and carbon and nitrogen material raw materials for RV’s adsorption, replication, and assembly. Simultaneously, RV can regulate the expression levels of intestinal glutamine transporters, GLS1, and glutamate transporters to entirely alter the metabolic flow and tissue distribution characteristics of L-glutamic acid in the host (Liu et al., 2024). RV can induce the large-scale migration and enrichment of L-glutamic acid from storage tissues such as skeletal muscle to the infection site of the intestinal tract. This abnormal redistribution of amino acids not only provides a core material basis for the continuous replication of the virus, but also causes intestinal metabolic disorders, epithelial cell function damage, and ultimately exacerbates intestinal inflammation and tissue damage (Liu et al., 2024). RV infection can significantly disrupt the metabolic homeostasis of glutamine-L-glutamic acid in host intestinal epithelial cells, driving the host metabolism to reshape in a direction conducive to virus replication. The intervention with ellagic acid can further decrease the serum L-glutamic acid level. Along with its potential anti-RV activity, it is speculated that it may inhibit the production of L-glutamic acid. The WB results show that compared with the model group, ellagic acid can significantly inhibit the expression of GLS1 in the jejunum. To probe the potential contribution of L-glutamic acid-related metabolism to the anti-RV effect of ellagic acid, we employed DON, a broad-spectrum glutamine antagonist that acts on multiple glutamine-dependent enzymes, to perturb glutamine-glutamate metabolic processes.

After treating RV-infected IEC-6 cells with DON and ellagic acid, the viral replication rate in the cells was significantly higher than that in the ellagic-acid-alone treatment group at both 24-hour and 48-hour time points. These observations support that perturbation of glutamine-glutamate metabolism can reverse the antiviral effect of ellagic acid and suggest that this metabolic axis may be involved in ellagic-acid-mediated anti-RV activity. Notably, because DON targets multiple glutamine-dependent enzymes, this rescue result alone cannot attribute the observed reversal specifically to GLS1 inhibition. Ellagic acid inhibits TLR4 and activates Nrf2, reducing L-Glu and the induced hippocampal nerve damage (Wang et al., 2020). The intestinal microbiota metabolite of ellagic acid, uric acid A (UroA), inhibits the activity of GLS1 to inhibit glutamine decomposition, alleviating portal hypertension in liver cirrhosis (CPH) (Li et al., 2024). Moreover, UroA can cross the blood-brain barrier and inhibit the activity of GLS1 in the brain, reducing glutamate synthesis and antagonizing excitatory neurotoxicity (Zhang et al., 2025). In conclusion, ellagic acid has the potential to regulate GLS1 activity and interfere with glutamate metabolism to inhibit RV replication. DON is a broad-spectrum glutamine antagonist that selectively inhibits all glutamine-dependent enzymes, with intestinal damage being the most prominent side effect (Grobben, 2024). Therefore, the phenomenon of virus replication being the most prominent after treatment with DON alone was observed. The antioxidant/anti-inflammatory effect of ellagic acid can partially alleviate the cell damage caused by DON, avoiding the severe metabolic collapse that cells may experience, as in the case of DON-alone treatment. Following DON exposure, EA failed to suppress RV replication and viral loads remained elevated. These findings support that the anti-RV activity of EA appears to be partially associated with intact glutamine-glutamate metabolism. Given the poly-target nature of DON, these data do not formally establish exclusive causality for GLS1.

Our earlier study confirmed that EA mitigates RV-caused intestinal damage through immune-microbiota regulation, which centered on inflammatory and gut microbial phenotypes without exploring host metabolic reprogramming. Distinct from the previous work, the present study reveals a novel GLS1-dependent glutamine-glutamate metabolic axis underlying EA anti-RV function. RV hijacks host glutamine metabolism by up-regulating GLS1 to generate L-glutamate for supporting viral replication. Via untargeted metabolomics and pharmacological perturbation with the broad-spectrum glutamine antagonist DON, our findings support that EA interferes with virus-associated glutamine-glutamate metabolism, in part through reducing GLS1 protein expression. This work establishes a metabolism-oriented mechanism for EA against RV and supplies new insights for developing metabolism-targeted natural anti-rotavirus therapeutics.

## 5 Conclusion

In summary, our untargeted metabolomics data indicated that rotavirus infection disturbs host glutamine-glutamate metabolic homeostasis in mice. RV-induced up-regulation of GLS1 protein was reversed upon ellagic acid administration. Pharmacological perturbation experiments using DON, a broad-spectrum glutamine antagonist, in IEC-6 cells suggested that glutamine-glutamate metabolism contributes to the anti-rotavirus effect of ellagic acid. Since DON acts on multiple glutamine-dependent enzymes, these results cannot uniquely assign the metabolic effect to GLS1, even though EA was observed to lower GLS1 protein levels. By suppressing GLS1, ellagic acid may interfere with virus-hijacked glutamine catabolism and reduce metabolic substrates required for rotavirus propagation.

## Data Availability

The datasets presented in this study can be found in online repositories. The names of the repository/repositories and accession number(s) can be found in the article/supplementary material.
